# Different Ability of Multidrug-Resistant and -Sensitive Counterpart Cells to Release and Capture Extracellular Vesicles

**DOI:** 10.3390/cells10112886

**Published:** 2021-10-26

**Authors:** Diana Sousa, Raquel T. Lima, Vanessa Lopes-Rodrigues, Esperanza Gonzalez, Félix Royo, Cristina P. R. Xavier, Juan M. Falcón-Pérez, M. Helena Vasconcelos

**Affiliations:** 1 i3S-Instituto de Investigação e Inovação em Saúde, Universidade do Porto, 4200-135 Porto, Portugal; dianaduartedesousa@gmail.com (D.S.); rlima@ipatimup.pt (R.T.L.); vanessa.rodrigues.85@hotmail.com (V.L.-R.); cristinax@ipatimup.pt (C.P.R.X.); 2 Cancer Drug Resistance Group, IPATIMUP—Institute of Molecular Pathology and Immunology of the University of Porto, 4200-135 Porto, Portugal; 3 Department of Biological Sciences, FFUP—Faculty of Pharmacy of the University of Porto, 4050-313 Porto, Portugal; 4 Department of Pathology, FMUP—Faculty of Medicine of the University of Porto, 4200-319 Porto, Portugal; 5 Cancer Signaling & Metabolism Group, IPATIMUP—Institute of Molecular Pathology and Immunology of the University of Porto, 4200-135 Porto, Portugal; 6 ICBAS-UP—Institute of Biomedical Sciences Abel Salazar of the University of Porto, 4099-003 Porto, Portugal; 7 Exosomes Lab. & Metabolomics Platform, CIC bioGUNE, CIBERehd, 28160 Derio, Spain; egonzalez@cicbiogune.es (E.G.); froyo.ciberehd@cicbiogune.es (F.R.); jfalcon@cicbiogune.es (J.M.F.-P.); 8 IKERBASQUE Basque Foundation for Science, 48013 Bilbao, Spain

**Keywords:** cancer multidrug resistance, extracellular vesicles, endocytic pathway

## Abstract

Cancer multidrug resistance (MDR) is one of the main challenges for cancer treatment efficacy. MDR is a phenomenon by which tumor cells become resistant to several unrelated drugs. Some studies have previously described the important role of extracellular vesicles (EVs) in the dissemination of a MDR phenotype. EVs’ cargo may include different players of MDR, such as microRNAS and drug-efflux pumps, which may be transferred from donor MDR cells to recipient drug-sensitive counterparts. The present work aimed to: (i) compare the ability of drug-sensitive and their MDR counterpart cells to release and capture EVs and (ii) study and relate those differences with possible distinct fate of the endocytic pathway in these counterpart cells. Our results showed that MDR cells released more EVs than their drug-sensitive counterparts and also that the drug-sensitive cells captured more EVs than their MDR counterparts. This difference in the release and capture of EVs may be associated with differences in the endocytic pathway between drug-sensitive and MDR cells. Importantly, manipulation of the recycling pathway influenced the response of drug-sensitive cells to doxorubicin treatment.

## 1. Introduction

Drug resistance is a major impediment for cancer treatment success [1]. Multidrug resistance (MDR) occurs in cells which become resistant to a variety of structurally and mechanistically unrelated drugs in addition to the drug initially administered [2]. Several mechanisms, such as increase in drug efflux, inhibition of apoptosis, alterations in cell cycle profile, or decrease in drug influx, may be responsible for the MDR phenotype [3]. Different studies have addressed not only the mechanisms involved in MDR but also how MDR may be disseminated to drug-sensitive cells, since impairment of the acquisition of the MDR phenotype is an important strategy to overcome this problem. 

The documented intercellular transfer of the drug-efflux pump P-glycoprotein (P-gp), mediated by extracellular vesicles (EVs) released by MDR cells and the subsequent acquisition of a MDR phenotype by the recipient sensitive tumor cells, was an important finding in this field of research [4]. Bebawy et al. investigated the potential involvement of EVs averaging a typical size of 500 nm in P-gp transfer. Drug-sensitive leukemia cells were co-cultured with EVs isolated from their drug-resistant counterparts. Following this incubation, the drug-sensitive cells showed presence of functional P-gp. The short time necessary for this effect (2 and 4 h) suggested that transfer of exogenous P-gp from donor (resistant) cells had occurred, rather than induction of transcription and subsequent translation of the MDR1 gene in recipient cells [5]. Since then, the EV-mediated transport of MDR players has been reported in a variety of studies [6,7,8,9,10]. The horizontal transfer of MDR phenotype to drug-sensitive cells has been evidenced by several of them [11,12,13].

Extracellular vesicles are membrane vesicles enclosed by a lipid bilayer, which contain cytosol from the donor (releasing) cells [14]. Their properties such as size, density, cargo or origin have been extensively studied in order to better understand their role under normal and pathological conditions [15]. Nevertheless, the high level of heterogeneity, both at the level of biogenesis and cargo loading, displayed by EV populations limits our understanding of their function [16]. Concerning the origin of EVs, the two main accepted processes are: (i) the outward budding and fission of the plasmatic membrane and (ii) the multivesicular body (MVB) formation along the endocytic pathway [14]. Several proteins were described as markers of these two processes; however, further studies showed that some of them were in fact shared between the two main biogenesis pathways [17]. Thus, the differentiation between the two pathways of EVs biogenesis is still very challenging. In the endocytic pathway, cargo is internalized from the plasma membrane and directed towards the early endosome, where it is sorted either towards (i) the late endosome or (ii) the recycling endosome. The content of the latter compartment is returned towards the plasma membrane. The cargo sorted towards the late endosome, instead, is mixed with other cargo of different origin and sorted in intraluminal vesicles (ILVs) through inward budding of the limiting membrane. At this stage the late endosome becomes a Multivesicular Body (MVB). These organelles can then fuse either with lysosomes, leading to cargo degradation, or with the plasma membrane, leading to release of ILVs in the extracellular space as EVs [18,19]. The maturation of the early endosome is tightly regulated and has been the object of extensive study [20]. 

In the present work, we show that the ability of drug-sensitive and their MDR counterpart cells to release and capture EVs is different. This difference is caused by differences in the fate of endocytic cargo in the two types of cells. Importantly, manipulating this pathway may be an important strategy to overcome MDR.

## 2. Results

### 2.1. MDR Cells Released More EVs Than Their Drug-Sensitive Counterparts and Showed Higher Levels of Rabs

**Figure 1:**
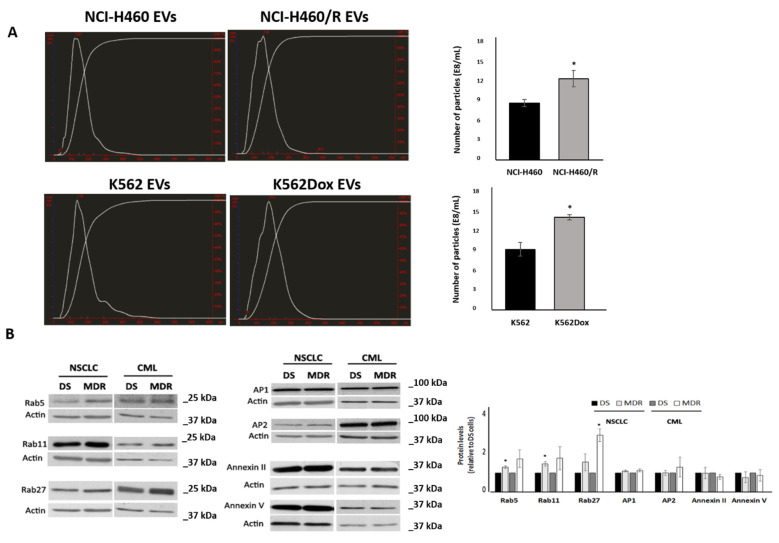
Analysis of EVs release by drug-sensitive (NCI-H460 and K562) and MDR (NCI-H460/R and K562Dox) counterpart cells. (**A**) Comparison of the number of EVs released, estimated by NTA measurements of EVs preparations. Left panel: NTA profile of EVs; right panel: quantification of number of particles normalised to the number of donor cells. * *p* ≤ 0.05. (**B**) Comparison of the levels of proteins associated to the release of EVs between drug-sensitive (DS: NCI-H460 or K562) and MDR (NCI-H460/R or K562Dox) counterpart cells, analysed by Western blot. Left panel: Representative blots from at least 3 independent experiments; right panel: densitometry analysis expressed after normalisation of the values obtained for each protein with the values obtained for actin (and further expressed in relation to drug-sensitive (DS) cells). * *p* ≤ 0.05.

Cancer drug-sensitive and their MDR counterpart cells (from both non-small cell lung cancer (NSCLC; NCI-H460 and NCI-H460/R) and leukaemia (K562 and K562DOX) models) were cultured in EVs-depleted medium during 72h. Their culture medium was then collected and EVs were isolated. EV size, morphology and classical markers were characterized according to previously published methods [21,22]. In our previous work, we found that MDR cells produced larger EVs than their drug-sensitive counterparts. In addition, EVs from MDR cells contained P-gp and presented a different content of proteins known to be involved in the biogenesis of EVs [22]. To quantify the number of particles released by drug-sensitive and their MDR counterpart cells, from both NSCLC and leukaemia models, nanoparticle tracking analysis (NTA) was performed in triplicate. In both models, the number of particles quantified by NTA was higher in the MDR cells than in their sensitive cell counterparts (NSCLC: 9.7 ± 0.53 vs. 12.4 ± 1.24; CML: 8.6 ± 1.03 vs. 14.1 ± 0.4). This difference was statistically significant (*p* ≤ 0.05) in both models studied (Figure 1A). 

A complex network of proteins has been described to regulate the release of EVs. Since we observed alterations between drug-sensitive and their counterpart MDR cells regarding their ability to shed EVs, the expression of some regulators of this mechanism was evaluated. Proteins from three different families, Rab family GTPase, annexin family and clathrin adaptor proteins (APs), were studied. The levels of these proteins were analyzed in the drug-sensitive and MDR cells from both NSCLC and CML models by Western blot and quantified by Quantity One. Importantly, the levels of some elements of the Rab family were found altered in MDR cells. In particular, Rab5, -11 and -27 were found increased in MDR cells in both models (Figure 1B) although this was only statistically significant for Rab5 and Rab11 in NSCLC cells and for Rab27 in CML cells (NSCLC: Rab5, 1.00 ± 0.0 vs. 1.31 ± 0.8, *p* ≤ 0.05; Rab11, 1.00 ± 0.0 vs. 1.49 ± 0.12, *p* ≤ 0.05; Rab27, 1.00 ± 0.0 vs. 1.58 ± 0.45; CML: Rab5, 1.00 ± 0.0 vs. 1.76 ± 0.46; Rab11, 1.00 ± 0.0 vs. 1.79 ± 0.61; Rab27, 1.00 ± 0.0 vs. 3.01 ± 0.33, *p* ≤ 0.05). Instead, the levels of AP1/2 as well as of Annexin II and -V were similar in drug-sensitive and MDR counterpart cells (Figure 1B).

### 2.2. Drug-Sensitive Cells Captured More EVs Than Their MDR Counterparts

**Figure 2:**
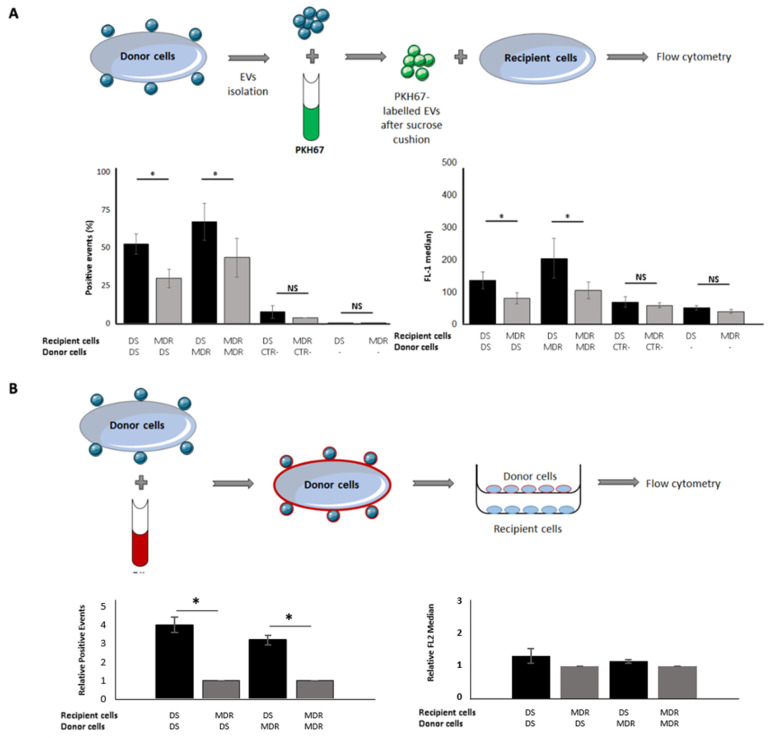
Comparison of EVs capture by drug-sensitive and MDR cells, using two approaches: PKH67 labelling of EVs (**A**) and DiI labelling of donor cells (**B**). (**A**) EVs isolated from K562 and K562Dox cells were labelled with PKH67 and incubated with recipient cells (K562 or K562Dox). Upper panel: experiment layout; lower panel: percentage of positive events and FL-1 median values. Results are presented as mean± SEM from three independent experiments. (**B**) Donor cells (K562 or K562Dox) were labelled with DiI and incubated with recipient cells (H460 or H460/R) using a trans-well system (0.4 µm). Upper panel: experiment layout; lower panel: percentage of positive events and PE-A median (relative to MDR cells as recipient). Results are presented as mean ± SEM from three independent experiments. * *p* ≤ 0.05, NS-non-significant.

To evaluate the ability of cells to capture EVs, two different approaches were used. In the first, EVs isolated from drug-sensitive and MDR counterpart cells (K562 and K562Dox, respectively) were labelled with the fluorescent membrane dye PKH67 [23]. Medium that had not been in contact with cells was also subjected to EVs isolation and PKH67 labelling, as negative control. Following a 30% sucrose cushion to eliminate dye aggregates, the labelled EVs were incubated with recipient cells and the amount of captured EVs determined by flow cytometry. Results showed that independently of the phenotype of the donor cells (drug-sensitive or MDR), the EVs’ capture was increased (*p* ≤ 0.05, n = 3) when the recipient cells were drug-sensitive (Figure 2A) (52.3% ± 11.4% vs. 29.8% ± 10.6%, *p* ≤ 0.05 and 67.3% ± 21.1% vs. 43.4% ± 22.1%, *p* ≤ 0.05). The EVs’ uptake was also slightly increased when the donor cells were MDR (52.3% ± 11.4% vs. 67.3% ± 21.1% and 29.8% ± 10.6% vs. 43.4% ± 22.1%). In the second approach, a porous trans-well system was used to evaluate the ability of recipient cells to capture EVs. The donor cells (K562 or K562Dox) were labelled with the long-term fluorescent dye DiI. DiI is weakly fluorescent until incorporation into membranes; therefore, all EVs released by donor cells were DiI-labelled. The labelled donor cells were placed in the upper part of a trans-well, and the recipient cells (NCI-H40 or NCI-H460/R) were placed in the bottom part of the trans-well (Figure 2B). Consistently with results obtained in the first approach, the EVs’ uptake by drug-sensitive cells was higher than that by MDR cells, independently of the type of (drug-sensitive or MDR) donor cell (4.0 ± 0.41 vs. 1.00 ± 0.0, *p* ≤ 0.05 and 3.18 ± 0.27 vs. 1.00 ± 0.0, *p* ≤ 0.05) (Figure 2B).

### 2.3. Fluid-Phase Endocytosis Was Higher in Drug-Sensitive Cells

**Figure 3:**
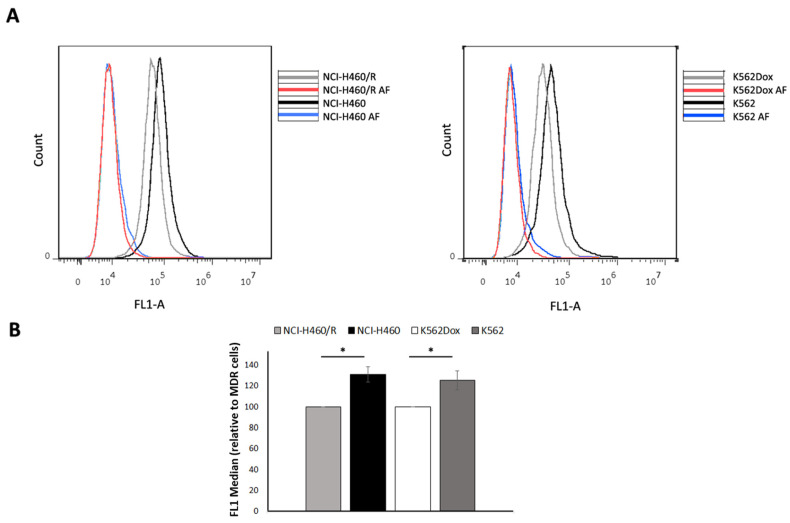
Comparison of the levels of fluid-phase endocytosis of dextran between drug-sensitive (NCI-H460, K562) and MDR (NCI-H460/R, K562Dox) counterpart cells, analyzed by flow cytometry. (**A**) Histograms representative of three independent experiments; (**B**) relative FL1 median of NCI-H460/R vs. NCI-H460 cells and of K562Dox vs. K562 cells. Data represent the mean ± SEM from three independent experiments. * *p* ≤ 0.05. Incubation of cells at 4 ℃ was performed as a negative control (ratio of FL1 median between DS vs. MDR cells: 97 and 106% for the NSCLC cells (NCI-H460/NCI-H460/R pair) and CML cells (K562/K562Dox pair), respectively). AF—autofluorescence.

We studied the endocytosis not mediated by receptor to compare this process with the internalisation of EVs in sensitive versus MDR cells. For this, the fluid-phase endocytosis of drug-sensitive and MDR cells, from both leukaemia and NSCLC models, was studied using the anionic fixable dextran (10,000 MW) [24,25]. Following 30 min at 37 C incubation of cells with 0.1 mg/mL of dextran, the abundance of incorporated dextran was quantified by flow cytometry (Figure 3). The results indicated a significant increase in the endocytosis rate of drug-sensitive cells (NCI-H460 and K562) when compared with the MDR counterpart cells (NCI-H460/R and K562Dox) (131 ± 7.4 vs. 100 ± 0.0, *p* ≤ 0.05 and 125.3 ± 9.0 vs. 100 ± 0.0, *p* ≤ 0.05) (Figure 3).

### 2.4. Drug-Sensitive Cells Showed Increased Expression of Flotillin-1

**Figure 4:**
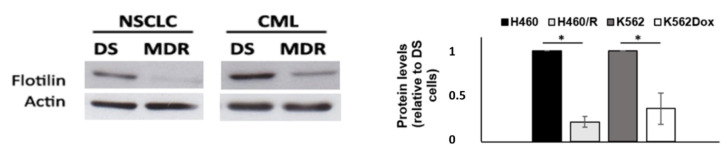
Analysis of expression of flotillin-1 in drug-sensitive and MDR counterpart cells. Comparison of the levels of flotillin-1 between DS (NCI-H460 or K562) and MDR (NCI-H460/R or K562Dox) counterpart cells, analyzed by Western blot. Left panel: representative images of blots from at least 3 independent experiments; right panel: densitometry analysis expressed after normalization of the values obtained for each protein with the values obtained for actin (and further expressed in relation to DS cells). Results represent the mean ± SEM from at least three independent experiments. * *p* ≤ 0.05.

The lipid content may influence the regulation of EVs uptake. Interestingly, drug-sensitive cells from both NSCLC and CML models showed higher levels of flotillin-1, a protein enriched in lipid rafts (NSCLC: 1.00 ± 0.0 vs. 0.19 ± 0.06, *p* ≤ 0.05; CML: 1.00 ± 0.0 vs. 0.32 ± 0.17, *p* ≤ 0.05) (Figure 4B). Preliminary data suggests an increase in bodipy staining in drug-sensitive cells (data not shown), therefore further studies should be performed to evaluate the role of lipid content in EVs uptake by drug-sensitive cells in these tumor models.

### 2.5. Drug-Sensitive and Their MDR Counterparts Presented Different a Fate of the Endocytic Pathway

**Figure 5:**
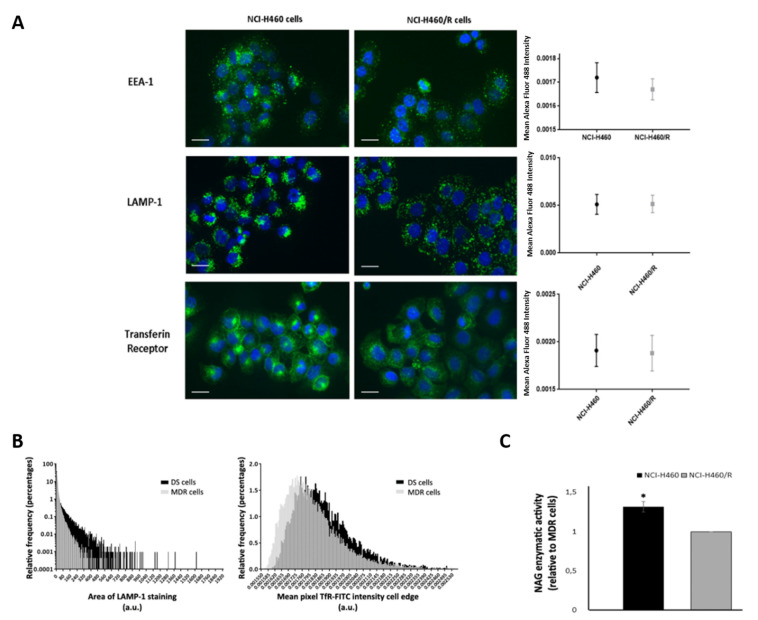
Analysis of the endocytic pathway in drug-sensitive (DS, NCI-H460) and MDR (NCI-H460/R) counterpart cells. (**A**) Comparison of the levels of expression of the endocytic markers EEA-1, LAMP-1 and transferrin receptor by immunofluorescence. Left panel: fluorescence microscopy of three independent experiments. Bar corresponds to 20 µm. Right panel: mean FITC intensity obtained for each protein following image analysis using ilastik and Cell Profiler™. Results represent mean ± SEM (n = 3). (**B**) Comparison of LAMP-1 intracellular distribution (left panel) and cell surface presence of transferin receptor (TfR) (right panel). Results obtained by immunofluorescence were quantified using the ilastik and Cell Profile™ software, to assess the cellular distribution of both proteins. Data correspond to the analysis of three independent experiments. (**C**) N-acetyl-D-glucosaminidase (NAG) enzymatic activity. Data were normalised for total protein content and further analysed in relation to MDR cells. Results are expressed as the mean ± SEM from three independent experiments. * *p* ≤ 0.05.

To evaluate the maturation from early (or sorting) endosome to late endosome, immunofluorescence for two proteins present in these structures (EEA-1 and LAMP-1, respectively [19]) was performed in the pair of adherent sensitive and counterpart MDR NSCLC cell lines. Drug-sensitive (NCI-H460) cells showed an increase in EEA-1 cellular expression (0.001720 ± 7.29 × 10^−7^ vs. 0,001670 ± 3.367 × 10^−7^, *p* ≤ 0.05). In contrast, no alterations in LAMP-1 expression were observed between drug-sensitive (NCI-H460) and MDR (NCI-H460/R) cells. Nevertheless, the intracellular distribution of LAMP-1 was different between the two cell lines (Figure 5A). Indeed, using cell image analysis software (ilastik and Cell Profiler™), the area of the LAMP-1 staining was quantified. In drug-sensitive cells, the patches of LAMP-1 staining were bigger, more typical of lysosomal distribution; in the MDR counterpart cells LAMP-1 was distributed as puncta, more typical of late endosome distribution (Figure 5B). To further study the increase in lysosomal pathway suggested by the LAMP-1 distribution in drug-sensitive cells, the activity of N-acetyl-D-glucosaminidase (NAG) was evaluated. NAG is a lysosomal enzyme responsible for degradation of glycolipids and glycoproteins [26]. In order to assess NAG activity, a specific enzymatic assay was performed. Drug-sensitive cells (NCI-H460) showed higher activity of NAG enzyme when compared with MDR cells (NCI-H460/R) (1.32 ± 0.07 vs. 1.00 ± 0.00, *p* ≤ 0.05) (Figure 5C). Results were normalized for protein content.

In addition, a marker of the recycling pathway, transferrin receptor [26] was also analyzed by immunofluorescence and quantified with the previously referred cell image analysis software. Drug-sensitive and MDR cells did not show differences in transferrin receptors expression (Figure 5A). Nevertheless, the machine learning tools indicated an increase in TfR cell surface expression in the drug-sensitive cells (Figure 5B). 

### 2.6. The Recycling Pathway Inhibitor, ONO-RS-082, Reduces the Growth Inhibition of Drug-Sensitive Cells following Treatment with Doxorubicin

**Figure 6:**
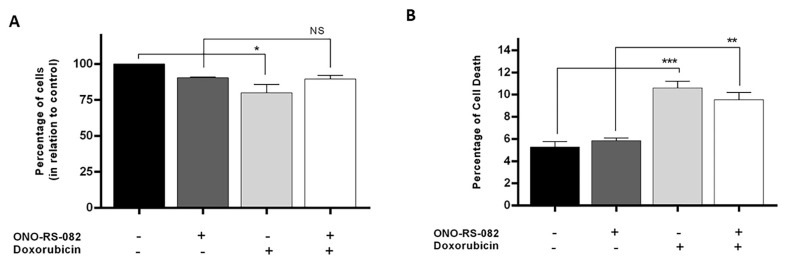
Analysis of the effect of a recycling pathway inhibitor (ONO-RS-082) on cell growth and cell death of NCI-H460 cells to doxorubicin, by SRB (**A**) and Annexin-V-FITC flow cytometry (**B**), respectively. Cells were pretreated for 16 h with ONO-RS-082 (2.5 µM) and further treated for 48 h with doxorubicin (4.7 nM in SRB and 20 nM in Annexin-V-FITC). Results were analysed in relation to control cells and are presented as the mean ± SEM from three independent experiments. * *p* ≤ 0.05, ** *p* ≤ 0.005, *** *p*≤ 0.0005, NS—non-significant.

The increase in transferrin receptor in the membrane of drug-sensitive cells suggests that the activation of the recycling pathway is higher in those cells when compared with MDR counterparts. Therefore, if the recycling pathway is related with drug resistance, inhibiting this pathway in those cells would result in alterations of their sensibility to doxorubicin. To evaluate the contribution of the recycling pathway to drug response, the NCI-H460 cells were pretreated with ONO-RS-082 for 16 h and then treated with doxorubicin (4.7 nM) for further 48 h. Importantly, at the time we added the drug, we also ensured that 2.5 µM of ONO-RS-082 was present in the cell culture medium in order to maintain the conditions. ONO-RS-82 was previously described as an inhibitor of the recycling pathway by inhibiting the phospholipase A2 activity [27]. After assessing the toxicity of ONO-RS-082 treatment alone using SRB assay (which caused a 10% decrease in cell growth), its effect in the cells’ sensitivity to doxorubicin was determined. Results showed that the growth inhibition of drug-sensitive cells following doxorubicin treatment was less pronounced when cells were pretreated with the recycling pathway inhibitor (ONO-RS-082) (100 ± 0.00 vs. 79.90 ± 5.95, *p* ≤ 0.05 and 90.38 ± 0.58 vs. 89.61 ± 2.42, *p* > 0.05) (Figure 6A). Similar results were observed using Annexin V-FITC staining assay. Indeed, cellular death induced by doxorubicin treatment was less pronounced when drug-sensitive cells were pretreated with the recycling pathway inhibitor (ONO-RS-082) (5.28 ± 0.37 vs. 10.60 ± 0.46, *p* ≤ 0.0005 and 5.84 ± 0.19 vs. 9.54 ± 0.51, *p* ≤ 0.005) (Figure 6B). 

## 3. Discussion

Chemotherapy efficacy is strongly impaired by MDR. Understanding how drug-sensitive cells acquire this phenotype is essential to overcome MDR. The horizontal transfer of MDR players between cancer cells has been described by several authors [28]. This is mediated by EVs released by donor MDR cells, which may be captured by recipient drug-sensitive cells. In the current study, we aimed to verify if drug-sensitive and their counterpart MDR cells were differentially prone to capture and release higher amount of EVs. Furthermore, we aimed to confirm if any differences found could be related to changes in the endocytic pathway. The increase in EVs release under stress/pathological conditions was already described in a variety of studies [29,30,31]. Our previous work indicated that there is an increase in EVs shedding by MDR cells of the tumor models studied in the present work: CML and NSCLC [32]; however, this observation needed to be further confirmed. Here, using NTA analysis, which allows the quantification of the number of particles, we confirmed that cells from two MDR cell lines (NCI-H460/R and K562Dox) shed higher amounts of EVs than their drug-sensitive counterparts (NCI-H460 and K562 cells). Importantly, previous work performed by others indicated that another population of EVs (isolated by using a lower centrifugation speed) was also released in higher amounts by MDR cells when compared with drug-sensitive counterparts [33]. Other studies that also referred this observation have suggested that the association of EVs release with drug resistance may be related to the calcium signaling as well as to the presence of ABC transporters [33,34,35,36]. Both tumor MDR cell models (NSCLC and CML) studied here overexpress P-gp, thus it is possible that P-gp (in addition to the Rab family) may be associated with increased EV release from MDR cells. Indeed, it is possible (biophysically) that the increase in P-gp (or other transmembrane proteins) creates a physical tension in the curvature of the cell surface, thus promoting increased EVs release. This hypothesis needs further testing.

A complex network of proteins have been described to regulate the release of EVs. Thus, the expression of proteins from three different families involved in this release process was studied. First, the levels of some Rab family GTPases were analyzed. As elegantly reviewed by Homma, Y et al., the Rab family of small GTPases comprises the largest number of proteins (∼60 in mammals) among the regulators of intracellular membrane trafficking, but the precise function of many Rabs and the functional redundancy and diversity of these proteins remain largely unknown [37]. Due to this, it was decided to assess the expression of some of the Rabs more studied among the EVs field, taking into consideration that we were studying two different tumor models: NSCLC (adherent epithelial cells) and CML (suspension cells). Interestingly, Rab 11 was previously described as important for modulation of the exosome pathway in K562 cells, although the exact step involved is still not known [38]. In addition, Rab 11 was also reported as an important player for tethering, docking and fusion of MVB, regulating exosome release as well as microvesicle budding in C. elegans and Drosophila [39]. Moreover, Rab5 was described as an essential modulator of early endosome maturation in adherent cells [18]. Rab27 is mostly localized in the late-endosome/lysosome-related organelles [14] and its knock-down resulted in a decrease in exosome release. In addition, an interesting work also indicated the association of Rab27 to microvesicle budding [40]. Secondly, we also studied the expression of some members of the Annexin family (Ca2^+^-binding proteins) which have also been described as important players in the budding process, regulating membrane dynamics. Annexin A2 was shown to induce membrane deformations, participating in the budding of viruses and in the formation of vesicles shed from cells [39]. Finally, we studied the levels of clathrin adaptor proteins (APs), usually associated with endocytosis due to cargo selection in clathrin-coated vesicles, but which may be also related to EVs’ release since they participate in the budding of viruses [41,42]. Our findings revealed that MDR cells have higher levels of Rab5 and Rab11 in the NSCLC model and higher levels of Rab27 in the CML model, indicating that the increased ability of MDR cells to produce and release EVs may involve the Rab family. However, further studies should be performed to assess this. Interestingly, different works have studied the impact of some members of the Rab family in the MDR phenotype: (i) Rab11, but not Rab4, was shown to facilitate cyclic AMP- and tauroursodeoxycholate-induced MRP2 translocation to the plasma membrane [43]; (ii) Rab27A and Rab27B downregulation enhanced sensitivity to cisplatin and induced apoptosis in ASPC-1 and PANC-1 cells [44]; (iii) Rab5A and Rab21 were found to play important roles in regulating ABCG2 surface localization and turnover [45]. Importantly, no involvement of AP1/2 or Annexin II/V was found.

The study of the interaction of EVs with recipient cells is still very challenging due to the: (i) high heterogeneity of isolated EVs (which may interact in a different manner with the recipient cells), and (ii) the variety of mechanisms involved in this process (e.g., membrane fusion or different types of endocytosis). The use of lipophilic dyes is frequent in such studies; however, interpretation of results should be cautious, since these dyes may form precipitates or may interact with wash buffers, producing aggregates. Alternatively, some studies described the genetic manipulation of donor cells in order to produce fluorescently tagged EVs-associated proteins (e.g., tetraspanins) which are sorted in EVs shed by these cells. However, the target protein must be carefully chosen if more than one condition is being studied, and its expression must not be altered between the different conditions under study. More complex techniques have also been developed, such as the use of multiplexed reporters [46]. Nevertheless, they are time-consuming and require the use of more technological advanced facilities. In the present study, in order to analyze the uptake of EVs by recipient cells, we used two fluorescent-based technologies: in the first approach, we labelled the isolated EVs; in the second, we labelled the donor cells. Taking into consideration the pitfalls described above, we performed a 30% sucrose cushion of the fluorescent-labelled EVs before their incubation with recipient cells, to eliminate artefacts. These strategies were efficient since the negative control (medium without cell contact, subjected to the same labelling procedure) showed lower signal when compared with conditioned medium. With both methods, we verified that drug-sensitive cells captured a higher amount of EVs than their MDR counterparts. Cell-specific EV uptake has been previously reported in other contexts. For example, Rana et al. described that tetraspanin 8-positive EVs shed by lymph node stromal cells were internalized more by endothelial and pancreatic cells than by parental cells [47]. Another study, carried out with EVs derived from rat pancreatic adenocarcinoma, concluded that subpopulations of leucocytes varied regarding exosome uptake; more EVs were captured by peritoneal exudate cells compared to granulocytes [48]. To our knowledge, this work presents the first evidence that drug-sensitive cells are more capable of capturing EVs than their MDR counterpart cells. 

In addition, we also observed an increase in EVs’ uptake when the donor cell was MDR. We would like to suggest that these two observations (increased EVs’ uptake by drug-sensitive recipient cells, particularly when with MDR donor cells), in association with the previously mentioned evidence that there is increased release of EVs by MDR cells, may have an important impact in the dissemination of MDR in cancer. Indeed, it is known that different players of MDR, such as drug-efflux pumps or non-coding RNAs, are present in EVs’ cargo. When the release of MDR cell EVs and uptake in DS cells is more efficient, the dissemination of a MDR phenotype may be more effective. EVs capture is triggered by different pathways, namely membrane fusion clathrin-mediated endocytois, caveolin-mediated endocytosis, phagocytosis, micropinocytosis and lipid raft-mediated endocytosis [49]. Using a 10,000 MW dextran, we confirmed that DS cells show an increased propensity for fluid-phase endocytosis. Importantly, in the present study we observed that both internalization processes (EV internalization and fluid-phase endocytosis) were shown to be increased in drug-sensitive cells. This indicates that endocytic processes, in general, seem to be more active in those cells. Additionally, the upregulation of EEA-1 (localized in a very early stage of early endosome [18]) observed in drug-sensitive cells suggested an increase in endocytosis, since endocytic vesicles merge with the early endosome [50]. Importantly, flotilin-1 was found upregulated in drug-sensitive cells from both NSCLC and CML models. Since flotillin is one of the constituents of lipid rafts [51,52], we hypothesize that in our studied models, the endocytosis of EVs may be mediated by flotillin-associated lipid rafts. However, to confirm this, further studies are required since the different lipid dynamics between MDR and DS cell lines here shown could be associated with general alterations in cellular metabolism under MDR conditions, as described by us in a previous work [53]. The analysis of caveolin levels showed no alterations between K562 cells and K562Dox cells and an increase in NCI-H460/R levels (justified by the effect of caveolin in drug resistance [54]) (Supplementary Figure S1). Importantly, it was described that lipid rafts which participate in EVs endocytosis may be independent of caveolin [49]. In addition, no alterations were observed in AP1/2 levels between drug-sensitive and MDR counterpart cells, also indicating that the endocytosis of these EVs was independent of clathrin. Nevertheless, we are not excluding the presence of the other aforementioned processes. Importantly, in the present work we did not evaluate other mechanisms such as endophilin-mediated endocytosis or macropinocytosis so, as previously mentioned, further studies are required. Different EVs’ capture pathways could occur at the same time in the same cell. In addition, it is important to note that we studied a highly heterogeneous EVs population, since we isolated them by differential ultracentrifugation without any concentration method. Moreover, some of the previous reported proteins are associated with more than one pathway.

**Figure 7:**
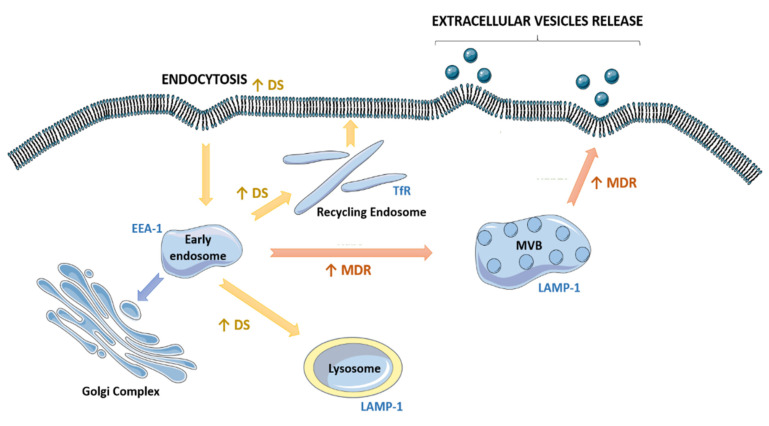
Schematic representation of the different fate of the endocytic pathway (EP) between drug-sensitive and MDR tumor cells. MVB—multivesicular body (late endosome). Blue font: classic markers of organelles; green font: Rab proteins family contribution to EVs release by endocytic and budding processes; yellow arrows: EP fate of drug-sensitive (DS) tumor cells; orange arrows: EP fate of MDR tumor cells.

The endocytosed content merges with early endosomes and is sorted for degradation, recycling or segregation. Previous reports showed that the majority of cargo internalized by ongoing endocytosis in mammalian cells is recycled back to membrane [55]. In drug-sensitive cells, transferrin receptor expression was increased at the cell surface, suggesting that these cells have an upregulation of the recycling pathway. In those cells, LAMP-1 localization indicated an increase in the lysosomal system, suggesting that the content not recycled to the membrane was degraded by lysosomes. Indeed, drug-sensitive cells showed higher activity of NAG enzyme (an enzyme associated to lysosome). In contrast, in MDR cells, the analysis of LAMP-1 distribution revealed the maturation of early endosomes to late endosomes. We suggest that the increase in the EVs released by MDR cells, previously quantified by NTA, is a consequence of this activation since late endosomes maturation is followed by EVs release (Figure 7). In the current work, only features related to the early, late and recycling endosomes were studied. The endosomal recycling compartment associated to the Golgi complex was not the focus of this study. 

The difference observed in the fate of the endocytic pathway between drug-sensitive and MDR cells seems to have an impact in drug response. Our results revealed that drug-sensitive cells treated with ONO-RS-082, a phospholipase 2 inhibitor which impairs the recycling pathway [32], altered the cellular response (in terms of cell growth and cell death) to doxorubicin. The alteration of the recycling pathway in MDR cells possibly results in a decrease in drug carriers and transporters at the cell surface, therefore decreasing the uptake of drugs. Indeed, different works have been trying to unravel the importance of the status of the endocytic pathway for a drug-resistant phenotype. Studies from Liang et al. reported that ERC alterations in drug-resistant cells influenced their response to drugs [56,57]. In addition, other authors also indicated that the endocytic pathway seems to be impaired in drug-resistant cells [58,59]. More recently, the impact of the endocytic pathway regulation in drug resistance mediated by EVs has been also assessed: Rab7A was shown to regulate cisplatin resistance by determining alterations in late endocytic trafficking and subsequent drug efflux through EVs [60]; chemotherapeutic agents were shown to stimulate the secretion and recycling of P-gp-enriched EVs through the dysregulation of endocytic pathway [61] and it was found that manipulation of the endocytic pathway attenuates extracellular vesicle-induced reduction in chemosensitivity to bortezomib in multiple myeloma cells [62]. Altogether, this evidence demonstrates the importance of unravelling the role of endocytic pathway regulation in drug resistance mediated by EVs. In our work, we suggest that the different fate of the endocytic pathway found in drug-sensitive and MDR cells may be related, at least in part, to the observed differences in the release and capture of EVs between MDR and drug-sensitive cells.

## 4. Materials and Methods 

### 4.1. Cell Culture

The following pairs of drug-sensitive and MDR counterpart cell lines were used: the NCI-H460 non-small cell lung cancer (NSCLC) cell line and the NCI-H460/R counterpart, which is MDR due to overexpression of P-gp (a kind gift of Dr. M. Pešić, Belgrade, Serbia) [63,64]; the K562 cell line derived from a chronic myeloid leukemia (CML) patient (ECACC) and the K562Dox counterpart which is MDR also due to P-gp overexpression (a kind gift of Dr. J.P. Marie, Paris, France) [65,66]. To preserve the MDR phenotype, 1 μM doxorubicin was added to the K562Dox cells every 2 weeks and 100 nM doxorubicin was added every month to the NCI-H460/R cells. Cell lines were genotyped and routinely monitored by PCR for possible mycoplasma contamination (Cell Culture and Genotyping Service, i3S). Cells were grown in RPMI-1640 medium with Ultraglutamine I and 25 mM HEPES (Lonza or Biowest) supplemented with 10% fetal bovine serum (FBS, Biowest) at 37C in a humidified incubator with 5% CO_2_ in air. Experiments were performed with cells in exponential growth and over 90% viability. 

### 4.2. Extracellular Vesicles Isolation

EVs-depleted FBS was obtained by ultracentrifugation of FBS overnight (at least 16 h). Cells were cultured in medium supplemented with 10% EVs-depleted FBS during 72 h. Following that period, the supernatant was collected and EVs were isolated by differential ultracentrifugation as previously described [21,22]. Briefly, EVs were isolated by various centrifugation steps of the supernatant at 4 °C: 10 min at 500× *g*, 10 min at 2000× *g*, 30 min at 10,000× *g* (high speed centrifuge AVANTI J-25, Beckman Coulter (Brea, CA, USA) and 60 min at 100,000× *g* (Optima XE-100 Ultracentrifuge, Beckman Coulter) to pellet the EVs, followed by one wash in PBS. EV pellets were processed according to different protocols as follows. Cellular viability was assessed by trypan blue exclusion assay prior the EVs isolation. EVs pellet was resuspended in PBS and processed according to different protocols, as follows. EVs characterization regarding size (by dynamic light scattering), morphology (by transmission electron microscopy), and presence of classical markers (by Western blot) was previously published [21,22]. 

### 4.3. Nanoparticle Tracking Analysis (NTA)

To assess the concentration of EVs released by MDR and drug-sensitive cells, NTA was used. First, protein was quantified using the Bio-Rad DC protein assay, according to the manufacturer’s instructions. Then, EVs were diluted in 500 µL PBS and analyzed in a NanoSight LM10 system (Malvern). Results for each sample were the average of at least 2 independent dilutions. Each dilution was analyzed twice. The post-acquisition settings, such as the camera level (12–15) or the screen gain, were the same for all samples. Results represent the mean ± SEM from at least three independent experiments. 

### 4.4. PKH67 EVs Labelling

To analyze the capture of EVs by recipient cells, the isolated EVs were labelled with PKH67 (Sigma) 1:500, according to the manufacturer’s instructions. Medium that had not been in contact with cells was also subjected to EVs isolation and PKH67 labelling, as negative control. In order to eliminate dye aggregates, the previously PKH67-labelled EVs were applied to a 30% sucrose cushion as described by Thery et al. [67]. These EVs were then incubated with recipient cells for 24 h. The same quantity of EVs was used for all incubations (inferred by protein quantification). The recipient cells were collected, washed in PBS, fixed with formaldehyde 2% and analyzed by flow cytometry using FACS Canto II (BD Biosciences), plotting at least 10,000 events per sample, after debris exclusion. The median results were obtained by the generation of histograms after debris exclusion. The percentage of positive events was obtained by the generation of PE-A versus FITC-A dot plots, using the autofluorescence of cells as background. Data analysis was performed using the FlowJo 7.6.5 software (Tree Star, Inc., Ashland, OR, USA) (Supplementary Figure S2).

### 4.5. DiI Staining

Another approach to evaluate the capture of EVs by recipient cells was used. In this second approach, donor cells were labelled with the fluorescent dye DiI 1:200 (Molecular Probes, Eugene, Oregon, USA) during 12 h at 37 °C. NSCLC recipient cells (50,000 cells/well) were seeded into a 12-well plate and the Trans-well Permeable Support, 0.4 µm poliester membrane (Costar) was placed above them (this pore size allow the passage of the majority of EVs studied in the present work). CML donor-labelled cells (500,000 cells/well) were placed on the top of trans-well. With this system, no direct contact between the drug-sensitive and MDR cells occurred. Following an incubation period of 48 h at 37 °C, cells were collected, washed in PBS, fixed with formaldehyde 2% and analysed by flow cytometry using FACS Canto II (BD Biosciences), plotting at least 10,000 events per sample, after debris exclusion. The median results were obtained by the generation of histograms after debris exclusion. The percentage of positive events was obtained by the generation of PE-A versus FITC-A dot plots, using the autofluorescence of cells as background. Data analysis was performed using the FlowJo 7.6.5 software (Tree Star, Inc. Ashland, OR, USA).

### 4.6. Fluid-Phase Endocytosis Assay

In order to analyze the ability of recipient cells for endocytosis, cells were incubated with 0.1 mg/mL anionic fixable dextran, Alexa Fluor™ 488; 10,000 MW (Thermo Fisher, Waltham, MA, USA) at 37 °C for 30 min. As negative control, dextran incubation was also performed at 4 °C. Cells were then washed in PBS and analyzed by flow cytometry using the BD Accuri™ C6 Flow cytometer, plotting at least 15,000 events per sample, after cell debris exclusion. Data analysis was performed using the FlowJo 7.6.5 software (Tree Star, Inc. Ashland, OR, USA).

### 4.7. Protein Expression Analysis

Cells were lysed in Winman’s buffer (1% NP-40, 0.1 M Tris-HCl pH 8.0, 0.15 M NaCl, and 5 mM EDTA) supplemented with EDTA-free protease inhibitor cocktail (Roche, Boulogne-Billancourt Cedex, France). Protein lysates were quantified with DC™ Protein Assay kit (Bio-Rad, Hercules, CA, USA) and 30 µg of protein were loaded on 12% sodium dodecyl sulphate polyacrylamide gel electrophoresis (SDS-PAGE) gel. After electrophoretic transfer into nitrocellulose membranes (GE Healthcare, Cleveland, OH, USA), the following primary antibodies, associated with EVs release and capture, were used: Rab5 (BD Biosciences, Erembodegem, Belgium).), Rab11 (BD Biosciences #610656, 1:500), Rab27 (R&D Systems #AF7245, 1:500), AP-1 (BD Biosciences #610386, 1:500), AP-2 (BD Biosciences #610502, 1:500), Flotillin-1 (Santa Cruz, Heidelberg, Germany, #74567, 1:100), Annexin II (Abcam, Cambridge, United Kingdom, #41803, 1:500) and Annexin V (Abcam #41196, 1:500). Then, the corresponding secondary antibodies were used: ECL Mouse IgG HRP-Linked whole Ab (Enzymatic, Portugal, 1:6000), ECL Rabbit IgG HRP-Linked whole Ab (Enzymatic, 1:6000), anti-mouse IgG-HRP (Santa Cruz, 1:2000), anti-rabbit IgG-HRP (Santa Cruz, 1:2000) or anti-goat IgG-HRP (Santa Cruz, 1:2000). Signal was detected using Amersham™ ECL Western blotting detection reagents (GE Healthcare), Amersham Hyperfilm ECL (GE Healthcare) and Kodak GBX developer and fixer (Sigma-Aldrich, St. Louis, MO, USA)). Data analysis was performed using the image software Quantity One—1D Analysis (Bio-Rad). Results represent the mean ± SEM from at least three independent experiments [68].

### 4.8. Immunofluorescence

In order to analyze the status of the endocytic pathway, the expression of classic markers of early endosome (EEA-1, BD 610457, 1:500), late endosome/lysosome (LAMP-1, Hibridoma Bank H4A3, 1:1000) and recycling endosome (transferin receptor, Hibridoma Bank B2/25, 1:300) was analyzed by immunofluorescence. NCI-H460 and NCI-H46/R cells were seeded on coverslips and grown to at least 80% confluence. Fixation was performed with 4% formaldehyde in PBS for 20 min, followed by washing and blocking in 0.1% BSA and 0.1% saponin in PBS for 30 min at room temperature. Cells were incubated with the following primary antibodies for 1 h at room temperature: transferin receptor (Hybridoma Bank), EEA-1 (BD Biosciences), LAMP-1 (Hybridoma Bank). The cells were further incubated with an Alexa Fluor 488 secondary antibody for 1 h. After washing, the coverslips were incubated with DAPI for 5 min, mounted and images were acquired on an IN Cell Analyser 2000 microscope (GE Healthcare) (focal length = 10.0; magnification = 20.0; numerical aperture = 0.45) using FITC and DAPI lasers (binning 1 × 1 and exposure: DAPI, 900,000 ms; FITC, 6,000,000 ms for TrfR, LAMP and 8,000,000 ms for EEA1) and processed with the ilastik and Cell Profiler™ software, as described below.

### 4.9. Fluorescence Quantification

For fluorescence analysis, we used two software: ilastik [69] and Cell profiler™ [70] For EEA-1 analysis, as we intend to analyze fluorescence intensity, Cell Profiler™ was used for nuclear segmentation followed by expansion; Alexa Fluor 488 intensity was then quantified. For LAMP-1 analysis, as we intended to analyze cellular distribution, ilastik was used for cell segmentation. Then, nuclear segmentation, expansion and puncta identification were performed, using Cell Profiler™. LAMP-1 staining area and Alexa Fluor 488 intensity were quantified in the overlay outlines. For transferrin receptor analysis, as we intended to analyze its presence at cell surface, first ilastik was used for cell segmentation. Afterwards, to delineate cells, nuclear segmentation and Alexa Fluor 488 propagation were performed using Cell Profiler™. Cell intensity edge and Alexa Fluor 488 intensity were then quantified in the overlay outlines. The layout of the image analysis using ilastik and Cell Profiler™ software is presented in Supplementary Figure S3.

For all analyses, a merge between overlays and original images was performed by Fiji/ImageJ, as quality control of the results. At least 3000 cells were analyzed for each replicate.

### 4.10. N-Acetyl-d-glucosaminidase (NAG) Activity

To analyze the lysosome activity, the enzymatic NAG assay was performed. Five hundred thousand cells/well were seeded and with 80% confluence, the same number of cells was lysed in lysis buffer (Tris-HCl 50 mM, pH 8.0, 0.5% Triton X-100). NAG activity was assessed using the β-N-Acetylglucosaminidase Assay Kit (Sigma) performed according to the manufacturer’s instructions. The results were normalized for total protein content (determined with the DC protein assay (Biorad).

### 4.11. Sulforhodamine B Assay

The Sulforhodamine B (SRB) assay was performed to evaluate the effect of ONO-RS-82 and doxorubicin treatment in the growth of NCI-H460 cells. Cells were seeded in 96-well plates (2500 cells/well), pretreated with ONO-RS-82 (2.5 µM) for 16 h and then treated in duplicate with doxorubicin (4.7 nM). Importantly, at the time we added the drug, we also ensured that 2.5 µM of ONO-RS-082 was present in the cell culture medium in order to maintain the conditions. NCI-H460 cells were treated with ONO-RS-82 only, and vehicle solvent as control. Following 48 h, cells were fixed in 10% (*w*/*v*) ice cold trichloroacetic acid (TCA), washed with distilled water and proteins were stained with 0.4% (*w*/*v*) SRB. Afterwards, cells were washed with 1% (*v*/*v*) acetic acid and the bound SRB was solubilized with 10 mM Tris base. Absorbance was measured at 510 nm in a multiplate reader (Synergy Mx, Biotek Instruments Inc.) [71]. The ratio between treated cells and the control cells was calculated.

### 4.12. Annexin V-FITC Flow Cytometry

The Annexin V-FITC Apoptosis Detection assay was performed to evaluate the effect of ONO-RS-82 and doxorubicin treatment in the cell death of NCI-H460 cells. Cells were seeded in 6-well plates (100,000 cells/well), pretreated with ONO-RS-82 (2.5 µM) for 16 h and then treated with doxorubicin (20 nM). Once again, at the time we added the drug, we also ensured that 2.5 µM of ONO-RS-082, was present in the cell culture medium in order to maintain the conditions. NCI-H460 cells were treated with ONO-RS-82 only, and vehicle solvent as control. Following 48 h treatment, cells were trypsinized and centrifuged, and then the pellet was re-suspended in binding buffer solution from the Annexin V-FITC Apoptosis Detection kit (eBioscience, Bender MedSystems), as indicated by the manufacturer’s instructions. The cells were then incubated for 10 min with 5 μL of human Annexin V-FITC and further incubated for 5 min more with 10 μL of propidium iodide (PI), in the dark and on ice. Samples were then analyzed by flow cytometry using the BD Accuri^TM^ C6 flow cytometer and the respective software, plotting at least 20,000 events per sample.

### 4.13. Statistical Analysis 

Data were statistically analyzed with the two-tailed Student’s *t*-test. Results were considered statistically significant when *p* ≤ 0.05. NS—non-significant.

## 5. Conclusions

The rate of processes such as proteins recycling to the cell surface or the release of EVs to the extracellular environment has an important impact on cellular homeostasis. Alterations in those processes can lead to aberrant protein localization and altered intercellular communication, which results in pathological conditions. Here, we found a natural ability of i) MDR cells to release higher amount of EVs than their DS counterpart cells and ii) drug-sensitive cells to capture more EVs than their MDR counterpart cells. We believe that this might have an impact on the MDR phenotype acquisition (by horizontal transfer) by drug-sensitive cells. A variety of previous studies described that MDR-derived EVs promote the acquisition of MDR phenotype by sensitive cells, by transferring different players of this phenotype to drug-sensitive cells. 

Based on the results here obtained, we argue that the different status of the endocytic pathway between MDR and drug-sensitive cells may explain, at least in part, our initial findings regarding differences in EVs release and capture by such cells. Importantly, an alteration of the recycling pathway reduced the cell growth inhibition and cell death induced by doxorubicin. Increasing knowledge about the players involved may contribute to identifying targets to overcome the horizontal transfer of MDR.

## Data Availability

The data presented in this study are available in article and supplementary material here.

## Supplementary Materials

The following are available online at https://www.mdpi.com/article/10.3390/cells10112886/s1, Supplementary Figure S1: Comparison of the levels of caveolin between drug sensitive (NCI-H460 or K562) and MDR (NCI-H460/R or K562Dox) counterpart cells, analyzed by Western blot. Supplementary Figure S2: Image representative of the FACS analysis. Supplementary Figure S3: Layout of the image analysis using ilastik and Cell Profiler™ software which allowed image cell segmentation and signal quantification.
