# Supplementary material for: Different Ability of Multidrug-Resistant and -Sensitive Counterpart Cells to Release and Capture Extracellular Vesicles
Source: Cells. 2021 Oct 26;10(11):2886. doi: 10.3390/cells10112886 (PMC8616370; doi:10.3390/cells10112886)
Supplement: Supplementary file 1 [file cells-10-02886-s001.zip › cells-1429213-supplementary.pdf]

Supplementary Materials:

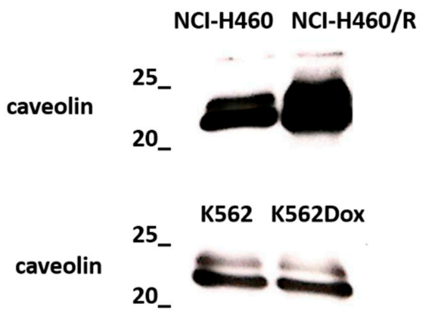

Supplementary Figure S1: Comparison of the levels of caveolin between drug sensitive (NCI-H460 or K562) and MDR (NCI-H460/R or K562Dox) counterpart cells, analysed by Western blot.

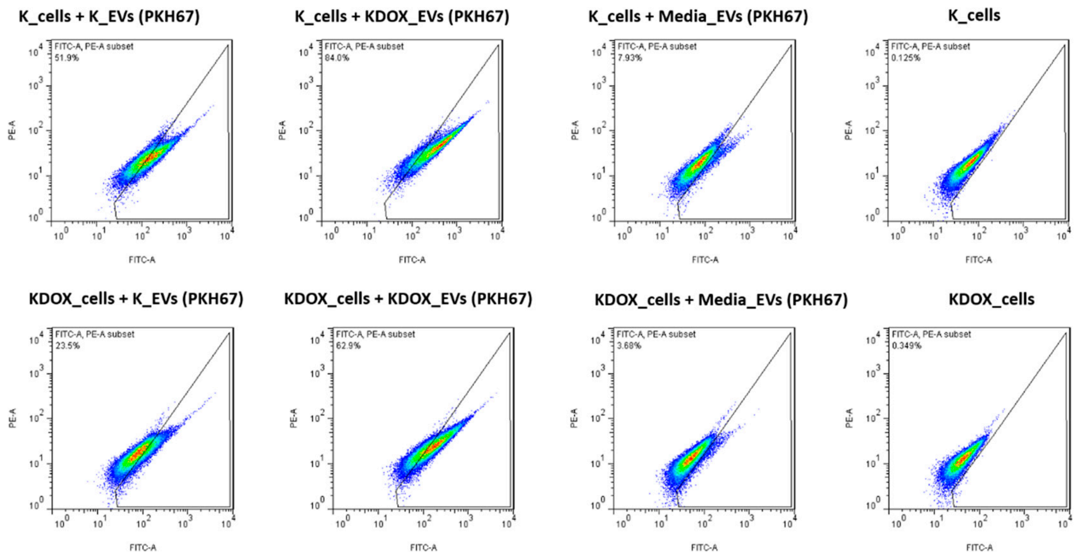

Supplementary Figure S2: Image representative of the FACS analysis.

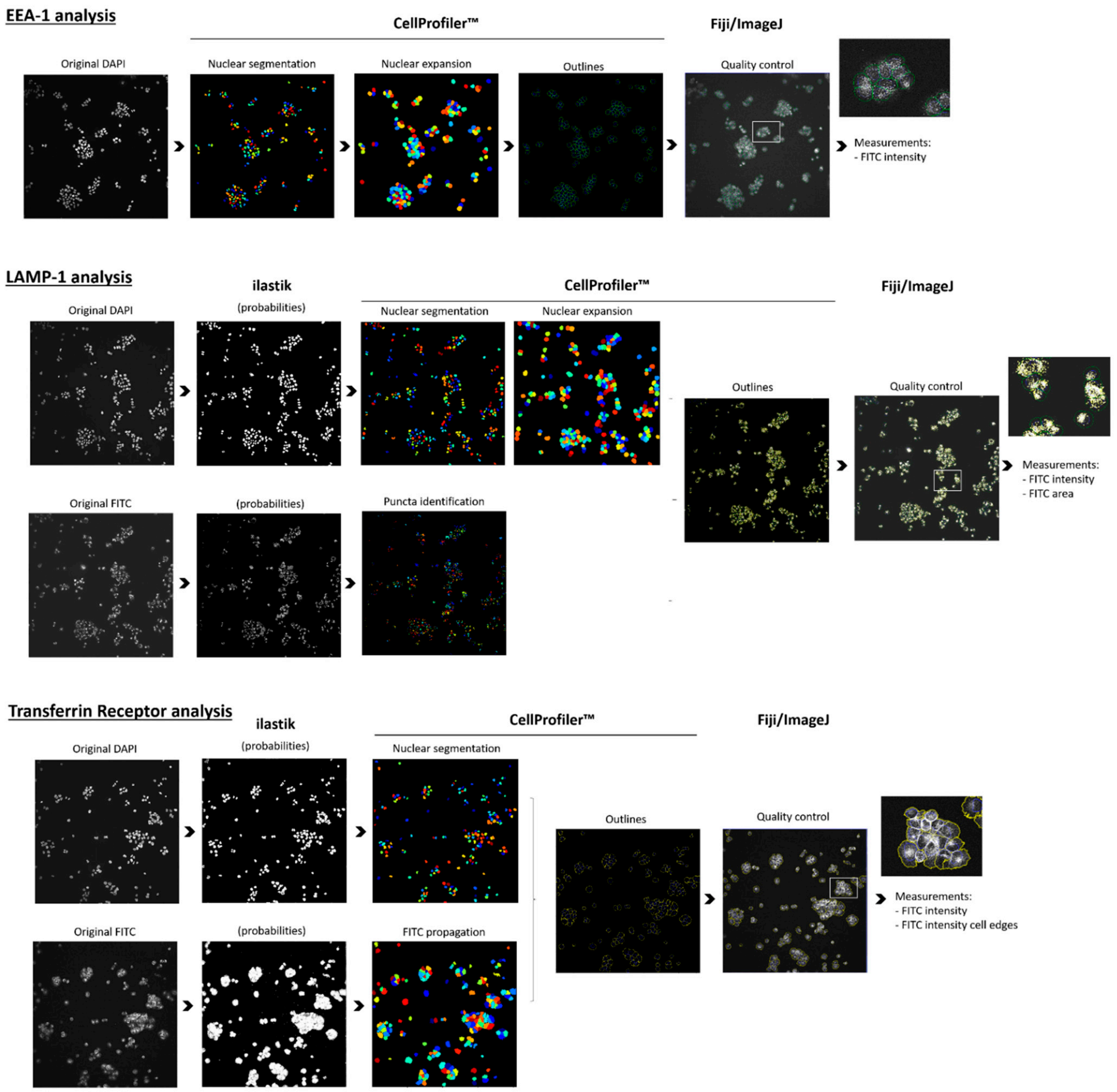

Supplementary Figure S3: Layout of the image analysis using ilastik and Cell Profiler™ software which allowed image cell segmentation and signal quantification.
